# Potential COVID-19 Drug Candidates Based on Diazinyl-Thiazol-Imine Moieties: Synthesis and Greener Pastures Biological Study

**DOI:** 10.3390/molecules27020488

**Published:** 2022-01-13

**Authors:** Sraa Abu-Melha, Mastoura Mohamed Edrees, Musa A. Said, Sayed M. Riyadh, Nadia S. Al-Kaff, Sobhi M. Gomha

**Affiliations:** 1 Department of Chemistry, Faculty of Science, King Khalid University, Abha 61413, Saudi Arabia; sabomlha@kku.edu.sa (S.A.-M.); mstorh@kku.edu.sa (M.M.E.); 2 Department of Organic Chemistry, National Organization for Drug Control and Research (NODCAR), Giza 12311, Egypt; 3 Department of Chemistry, Faculty of Science, Taibah University, Al-Madinah Al-Munawarah 30002, Saudi Arabia; riyadh1993@hotmail.com; 4 Department of Chemistry, Faculty of Science, Cairo University, Giza 12613, Egypt; 5 Department of Biology, Faculty of Science, Taibah University, Al-Madinah Al-Munawarah 30002, Saudi Arabia; nkaff@taibahu.edu.sa; 6 Department of Chemistry, Faculty of Science, Islamic University of Madinah, Al-Madinah Al-Munawarah 42351, Saudi Arabia

**Keywords:** imines-tethered thiazoles, synthesis, COVID-19, in silico molecular docking, molnupiravir, remdesivir, greener pastures

## Abstract

A novel series of 1-aryl-*N*-[4-phenyl-5-(arylazo)thiazol-2-yl)methanimines has been synthesized via the condensation of 2-amino-4-phenyl-5-arylazothiazole with various aromatic aldehydes. The synthesized imines were characterized by spectroscopic techniques, namely ^1^H and ^13^C-NMR, FTIR, MS, and Elemental Analysis. A molecular comparative docking study for **3a–f** was calculated, with reference to two approved drugs, Molnupiravir and Remdesivir, using 7BQY (M^pro^; PDB code 7BQY; resolution: 1.7 A°) under identical conditions. The binding scores against 7BQY were in the range of −7.7 to −8.7 kcal/mol for **3a–f**. The high scores of the compounds indicated an enhanced binding affinity of the molecules to the receptor. This is due to the hydrophobic interactions and multi-hydrogen bonds between **3a–f** ligands and the receptor’s active amino acid residues. The main aim of using in silco molecular docking was to rank **3a–f** with respect to the approved drugs, Molnupiravir and Remdesivir, using free energy methods as greener pastures. A further interesting comparison presented the laydown of the ligands before and after molecular docking. These results and other supporting statistical analyses suggested that ligands **3a–f** deserve further investigation in the context of potential therapeutic agents for COVID-19. Free-cost, PASS, SwissADME, and Way2drug were used in this research paper to determine the possible biological activities and cytotoxicity of **3a–f**.

## 1. Introduction

Since the appearance of COVID-19, followed by the Delta variant and recently the potentially more infectious Omicron strain pandemic caused by severe acute respiratory syndrome, the world has taken action for protection, such as emphasizing the importance of social distancing/masking [1,2]. However, the virus is still spreading scarily and has caused extensive social, economic, and health disorders. Hence, efficient drugs are instantly needed to end this pandemic and help the society return to routine. The good news is that various COVID-19 vaccines have been developed and evaluated at an extraordinary speed [3,4,5,6,7,8]. Having this in view, in this paper, we will test and evaluate our compounds by comparing their performances to the two well-established drugs (Molnupiravir and Remdesivir). An in silco method will be employed as a preliminary study to filter and rank our compounds as greener pastures at this stage [9,10,11].

Furthermore, urgent needs for novel antimicrobial and anticancer agents have directed many researchers to design and synthesize a variety of bioactive heterocycles. Thiazoles are incorporated in most drugs such as Sulfathiazole (antimicrobial), Tiazofurin (antineoplastic), Ritonavir (antiretroviral), and Abafungin (antifungal) [12]. In addition, imine-containing azoles are highlighted to develop new potential drug candidates with diverse therapeutic efficacy such as anticancer, antibacterial, antifungal, antioxidant, antimicrobial, and antiproliferative activities [13,14,15,16]. Moreover, Schiff’s bases have gained prominence role for antimicrobial, antituberculosis [17], anticonvulsant, antidepressant, analgesic, anti-inflammatory, antitumoral, vasodilator, and antiviral [18] activities. The synergism of thiazole with imine moiety in a hybridized molecule may result in increasing the biological potency, mainly because of the variation of substituents in the imine derivatives. Electron-donating and electron-withdrawing groups on arylazo moiety may affect biological activities. Promoted by the above observations, it was aimed to synthesize 1-aryl-*N*-[4-phenyl-5-(arylazo)thiazol-2-yl)methanimines and evaluate their biological importance. It is important to mention here that we used 7BQY (M^pro^, PDB) over 6LU7, because the crystal structure of the COVID-19 main protease in complex with the inhibitor, N3, has a resolution of 1.7 Å whereas 6LU7 is with a resolution of 2.16 Å. Furthermore, this study aimed to rank the ligands with respect to two approved and widely used drugs.

## 2. Results and Discussions

**Scheme 1:**
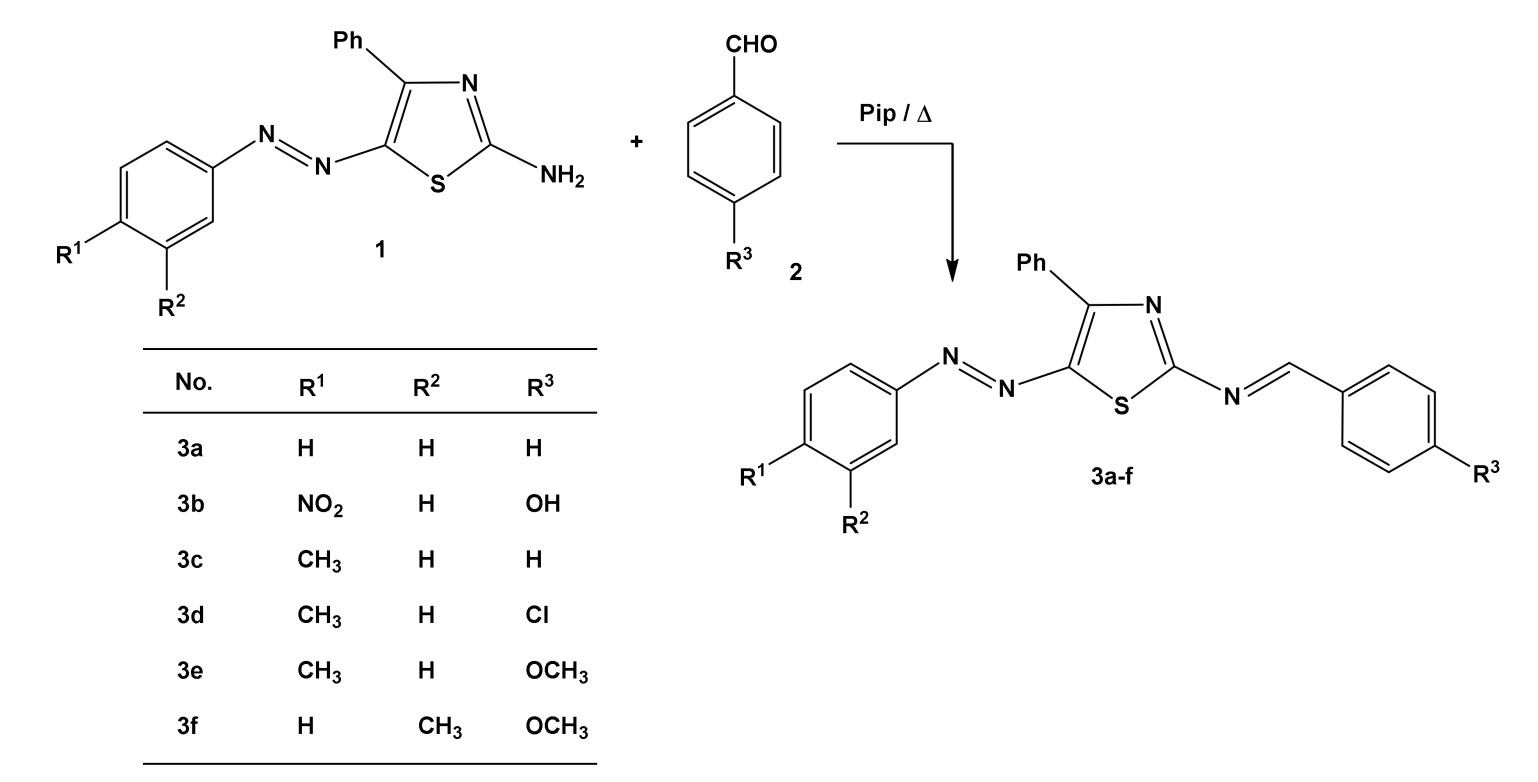
Synthesis of arylazothiazolimines **3a–f**.

The condensation reaction is an endeavor to synthesize imine compounds. Thus, the condensation of 2-amino-4-phenyl-5-arylazothiazole (**1**) with various aromatic aldehydes **2** in boiling ethanol and catalytic drops of piperidine resulted in the formation of the respective imine derivatives **3a–f,** with electron-donating and electron-withdrawing groups on arylazo moiety (Scheme 1).

**Chart 1:**
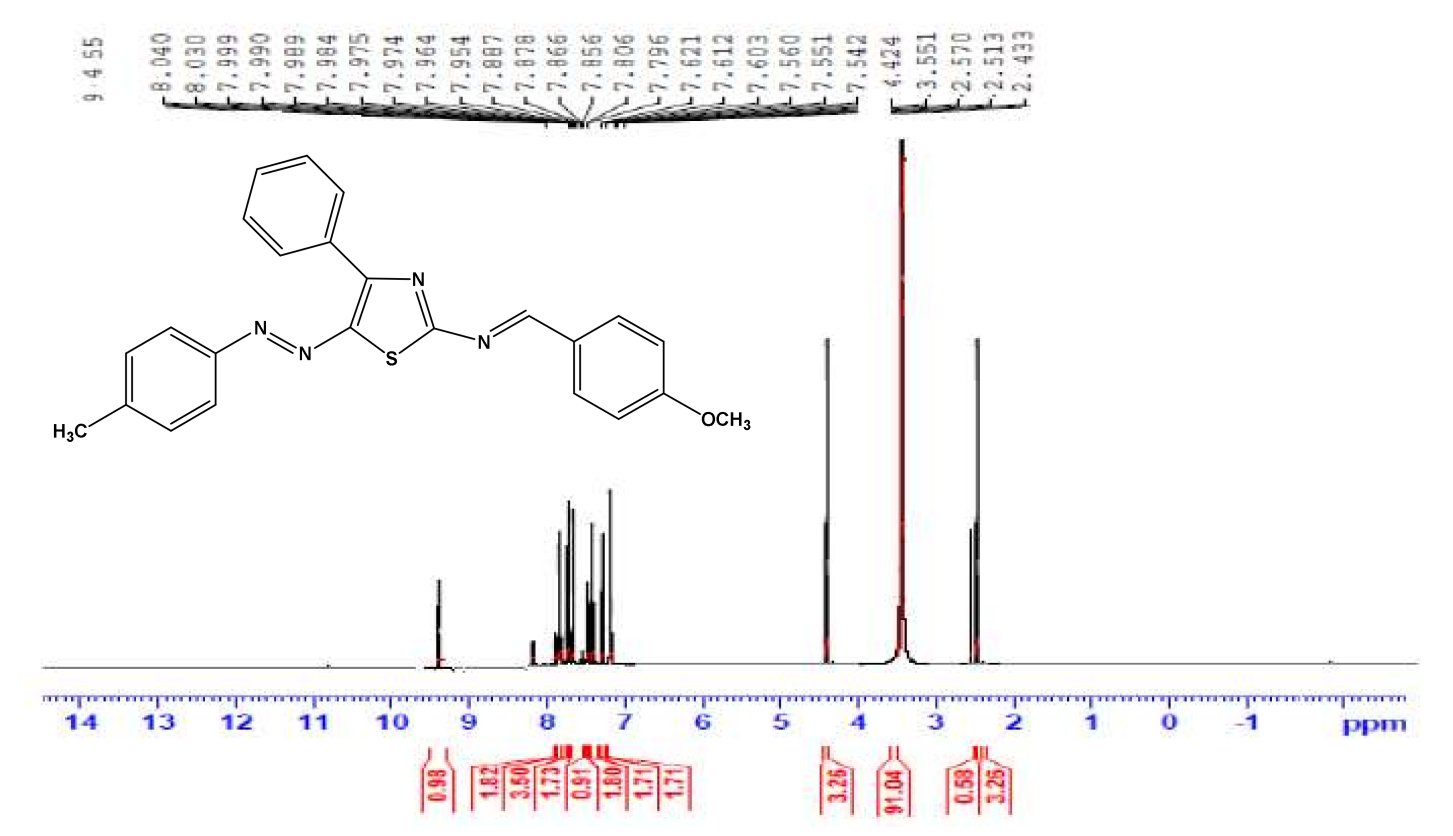
^1^H-NMR of compound **3e** as a representative example. More spectra are available on line as Supplementary Materials: Figure S1: IR of compound (**3a**), Figure S2: ^1^H-NMR of compound (**3a**), Figure S3: ^13^C-NMR of compound (**3a**), Figure S4: MS of compound (**3a**), Figure S5: IR of compound (**3e**), Figure S6: ^1^H-NMR of compound (**3e**), Figure S7: ^13^C-NMR of compound (**3e**), Figure S8: MS of compound (**3e**).

The isolated products gave analytical data consistent with the imine structures. ^1^H-NMR revealed one singlet signal at *δ* = (9.41–9.51) ppm due to HC=N (Chart 1) [19]. The molecular ion peaks and microanalysis results were also in accordance with theoretical calculations for imine products.

### 2.1. Molecular Modeling against 7BQY

**Figure 1:**
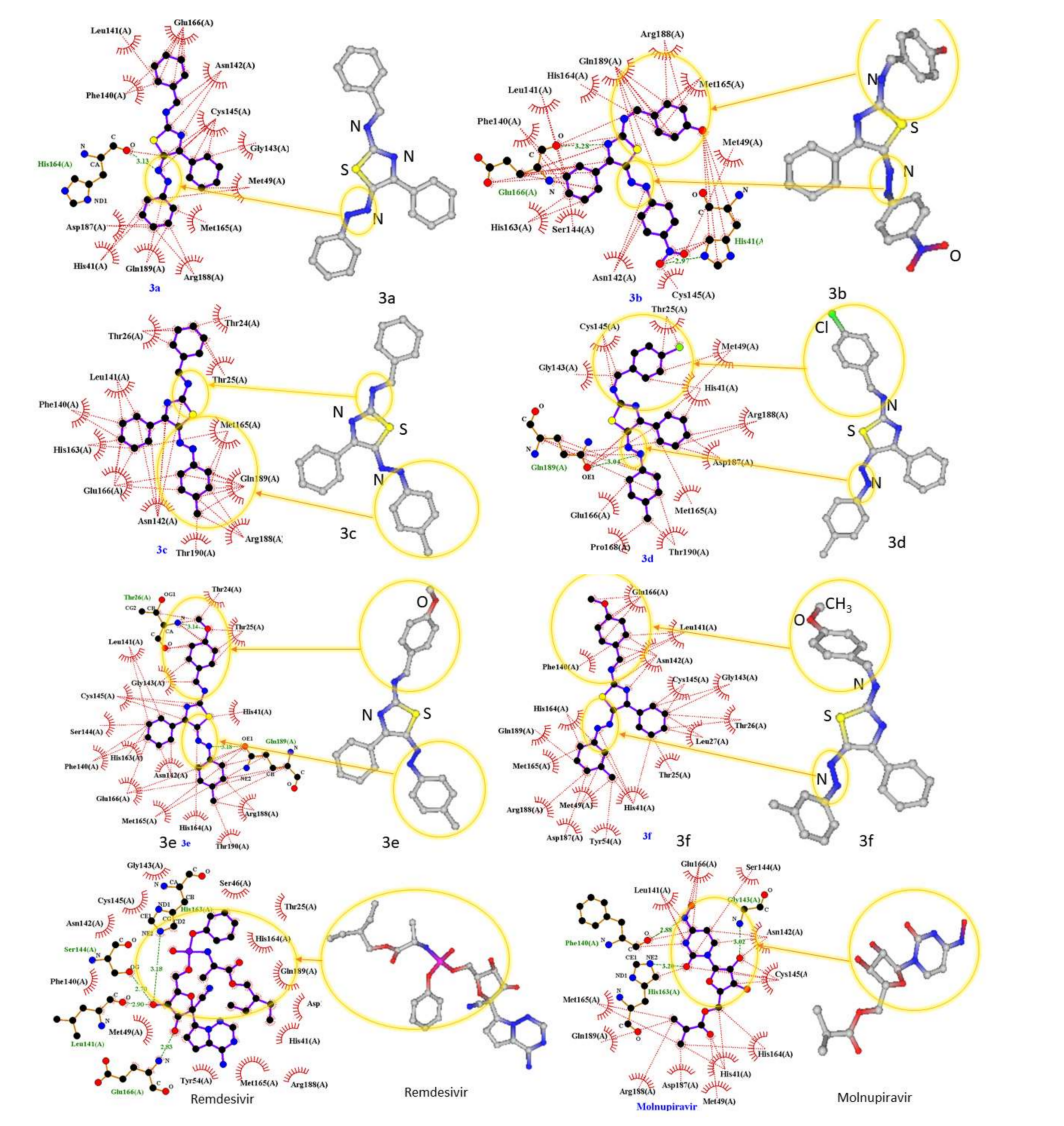
A schematic 2D LIGPLOT (LIGPLOT software is a program to generate schematic diagrams of protein-ligand interactions) representation showing **3a–f**, Remdesivir, and Molnupiravir before and after docking into the binding pocket of 7BQY. The variations in the ligands are highlighted by yellow circles.

**Figure 2:**
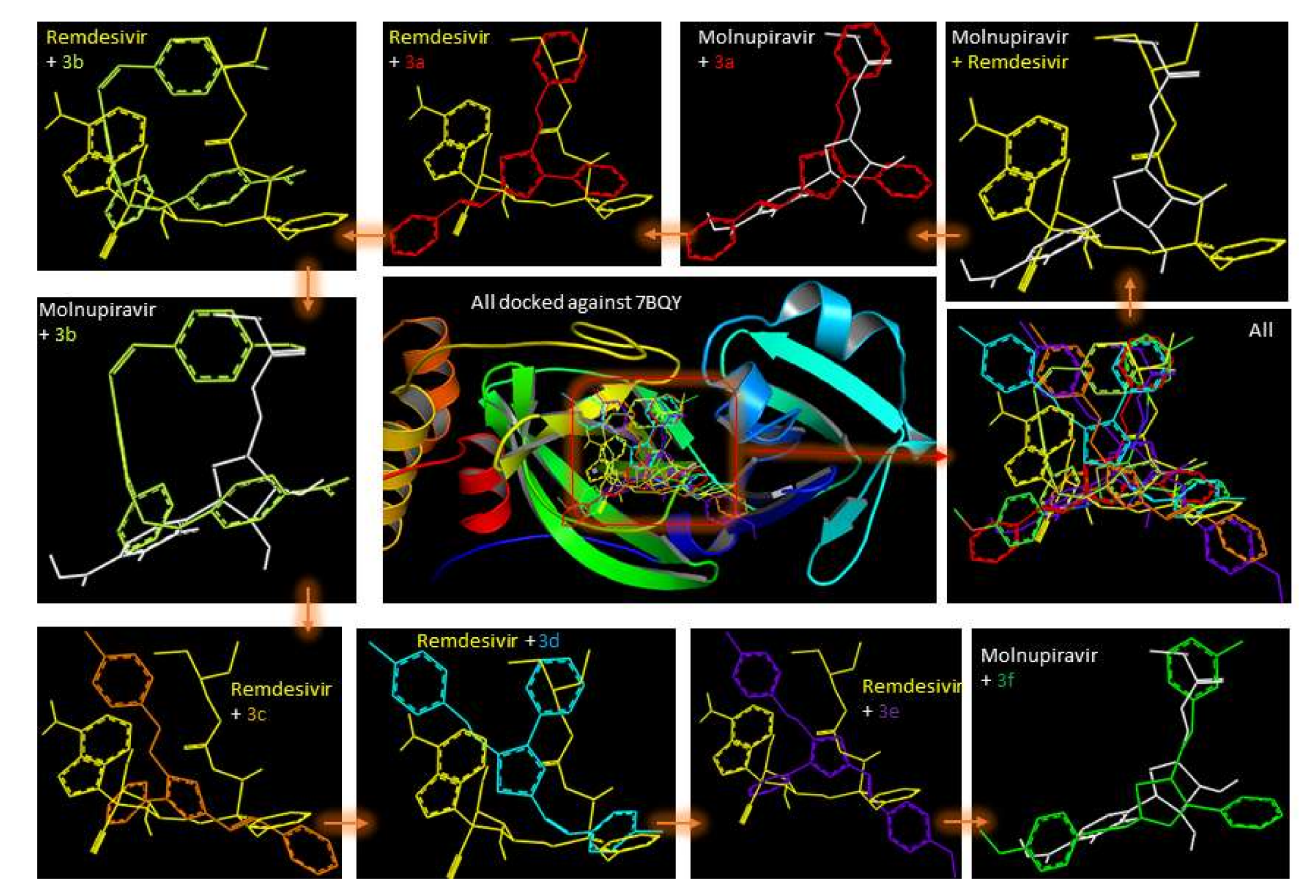
The superpositions of compounds **3a–f** and the approved drugs docked together into the binding pocket of 7BQY. Ligands, drugs, and labels are color-coded.

The world is experiencing a tough time at many economic and social activities worldwide. It has been hit severely by COVID-19 and many variants, including the new Omicron variant [20,21,22]. Hence, we have been encouraged to study how ligands **3a–f** might behave in the main protease’s active site for COVID-19 (M^Pro^; PDB code: 7BQY) compared to the approved inhibitors, Molnupiravir and Remdesivir, in an effort to contribute to solving the outbreak of the pandemic disease. In this study, we preferred to use the 7BQY, because it has been docked against the well-known inhibitor N3 that appeared recently in Nature journal [23]. In addition, both the approved drugs are known to block the activity of RNA-dependent RNA polymerase (RdRp). However, they also affect viral replications through M^pro^ activity [24,25]. It is vital to be armed against viral protein to hinder its activities by inhibiting more than one of its proteins. Therefore, we carried out this study using a computer-assisted method to filter the compounds ahead to any in vivo and other experiments to minimize energy wastage following the recommendation from (COP26, in Glasgow on 31 October–13 November 2021). In actuality, computational science techniques and software are now at an excellent availability and an accuracy stage, making computational experiments more feasible than before for the drug design business. It is interesting to know that computer-aided drug designs have been in use for more than 40 years [26,27]. This study performed the binding affinities for ligands **3a–f** with 7BQY. In this computer-aided work, PyRx software (version 0.8) [28] considered 7BQY a flexible protein and **3a–f**, Remdesivir, and Molnupiravir rigid ligands. However, the displays of the ligands before and after docking into 7BQY were not identical in all docking cases (Figure 1). For example, the N=N bond in ligand **3a** was oriented opposite to **3a** in the protein cavity of 7BQY, which may be due to the H-bond with His164(A) (Figure 1), indicating ligand **3a** after docking in the protein cavity did not stay the same as it was before docking. The superpositions of compounds **3a–f** (color-coded), Molnupiravir, and Remdesivir (approved drugs by the FDA [29,30]) were docked against 7BQY using the same parameters for a comparison (Figure 2).

The results were represented by PyMOL [31] in the square frame (Figure 2). For the orientation comparison, each rectangular presents a ligand superimposed over either Remdesivir or Molnupiravir.

**Figure 3:**
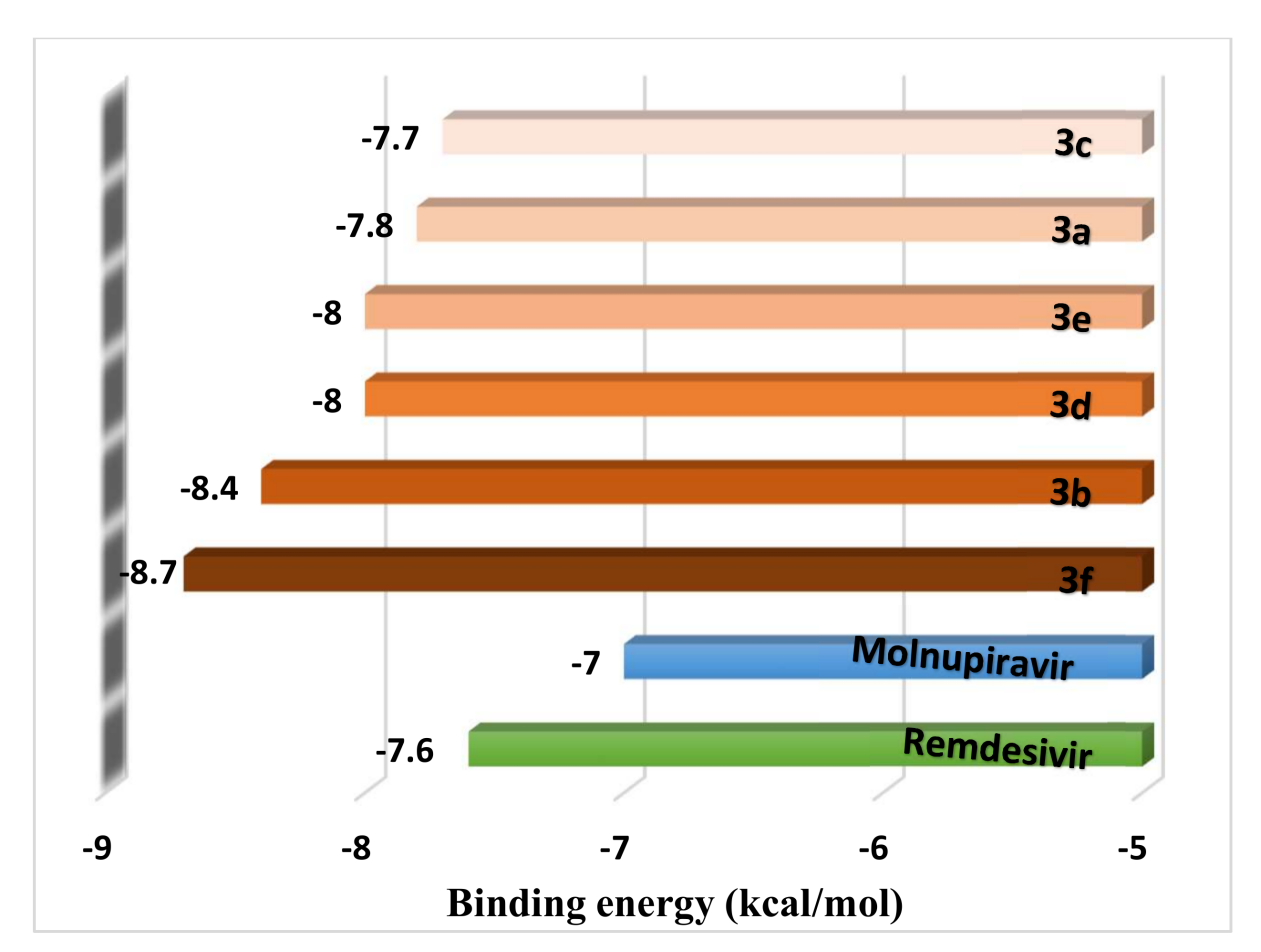
Binding affinities of compounds **3a–f** against the COVID-19 protease 7BQY and the approved medicines for a comparison.

The superpositions revealed a close overlap between the ligands and either of the drugs in the protease site. Furthermore, the binding energies of Molnupiravir and Remdesivir were −7.0 and −7.6 kcal/mol, respectively. Compounds **3a–f** showed binding energies in a range of −7.7 and −8.7 kcal/mol, indicating possible similar biological activities to the two drugs [32,33]. This may suggest a further possible study of compounds **3a–f** in the context of a potential medicine for COVID-19 is recommended. The reason for choosing these approved drugs is that they are active against different viral diseases, including influenza [32]. It is essential to mention that all the docked molecules against the target enzyme COVID-19 were ranked according to their binding energies, shown in ascending order and color-coded molecules (Figure 3).

**Figure 4:**
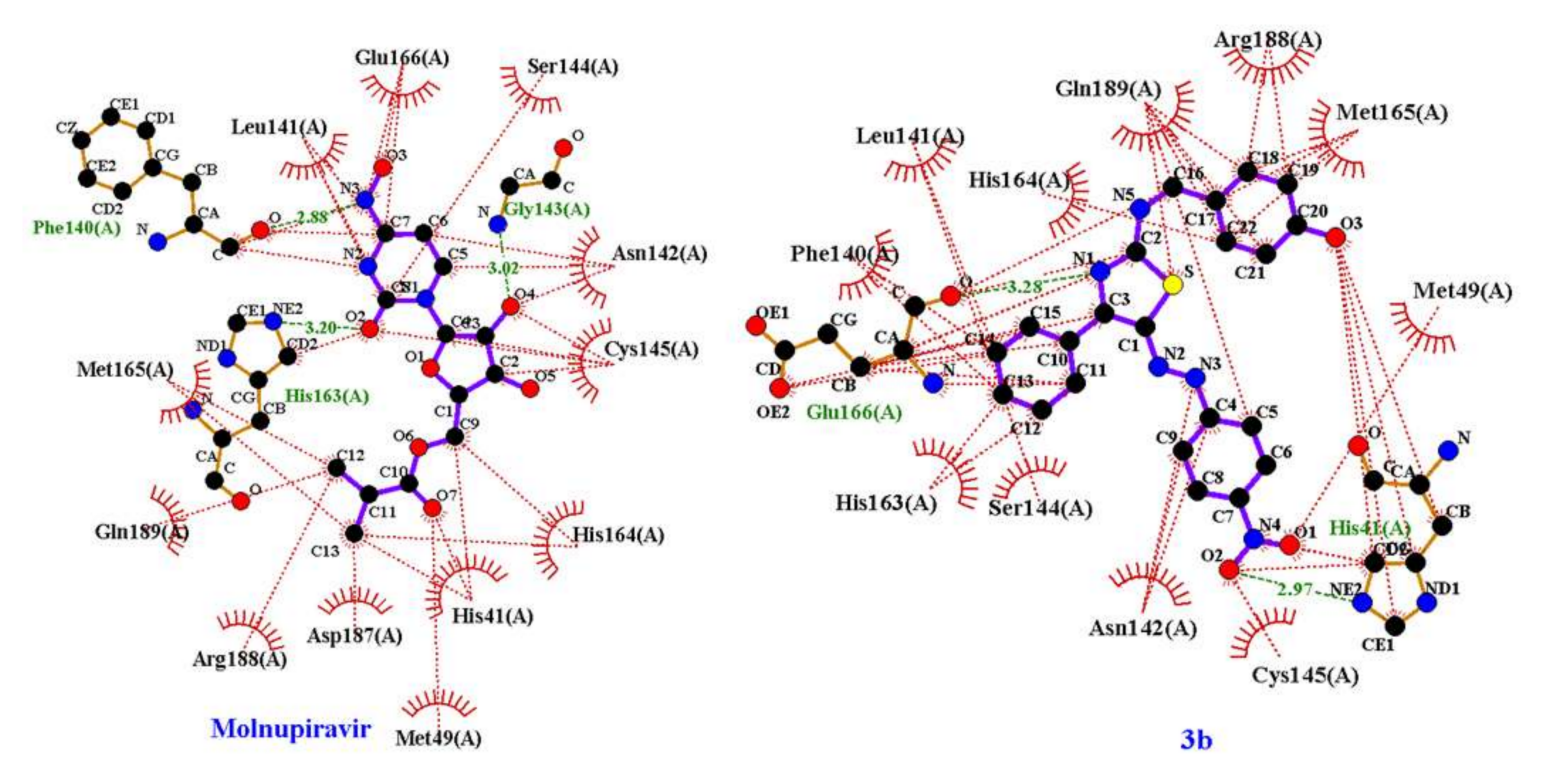
A schematic 2D LIGPLOT representation of Molnupiravir and **3b** against the complex 7BQY showing the hydrogen and hydrophobic interactions.

The binding affinity was attributed to many hydrogen bonds (green lines) and hydrophobic interactions (red lines) between ligands **3a–f** and the receptor’s active amino acid residues, as shown in Schematic 2D LIGPLOT (Figure 1 and Figure 2). It could be seen that **3b** formed two H-bonds with Glu166 and His41(A), whereas Molnupiravir formed three H-bonds with Phe140(A), His163(A), and Gly143(A), (Figure 4). Detailed key symbols can be found in a recent report [34].

**Figure 5:**
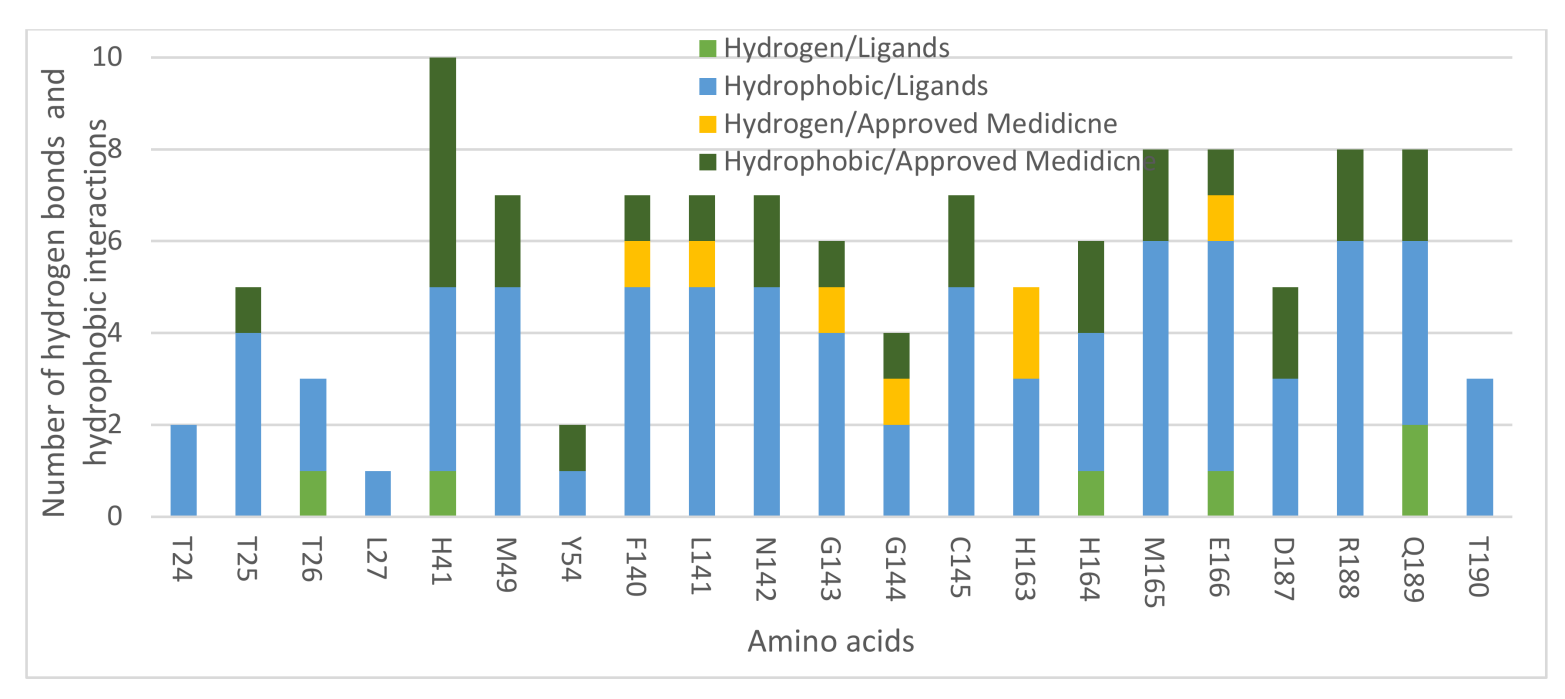
The numbers of hydrophobic interactions and H-bonds revealed upon docking 7BQY protease against compounds **3a–f** and the approved drugs.

All interactions were summarized for compounds **3a–f** and the approved drugs in Figure 5.

The box plot produced with Minitab 19 presented the number of hydrogen and hydrophobic interactions between the amino acid residues of the M^pro^ substrate of COVID-19 (7BQY) and compounds **3a–f** in comparison to the two approved medicine. One of the approved drugs is already known and developed to treat Ebola virus cases infection by Gilead Sciences Inc., (Foster City, CA, USA) [35,36]. Recently, Molnupiravir [37] was approved to treat COVID-19 orally [38]. It is a broad-spectrum ribonucleoside inhibitor of RdRp RNA influenza and respiratory syncytial viruses such as COVID-19 [37].

**Figure 6:**
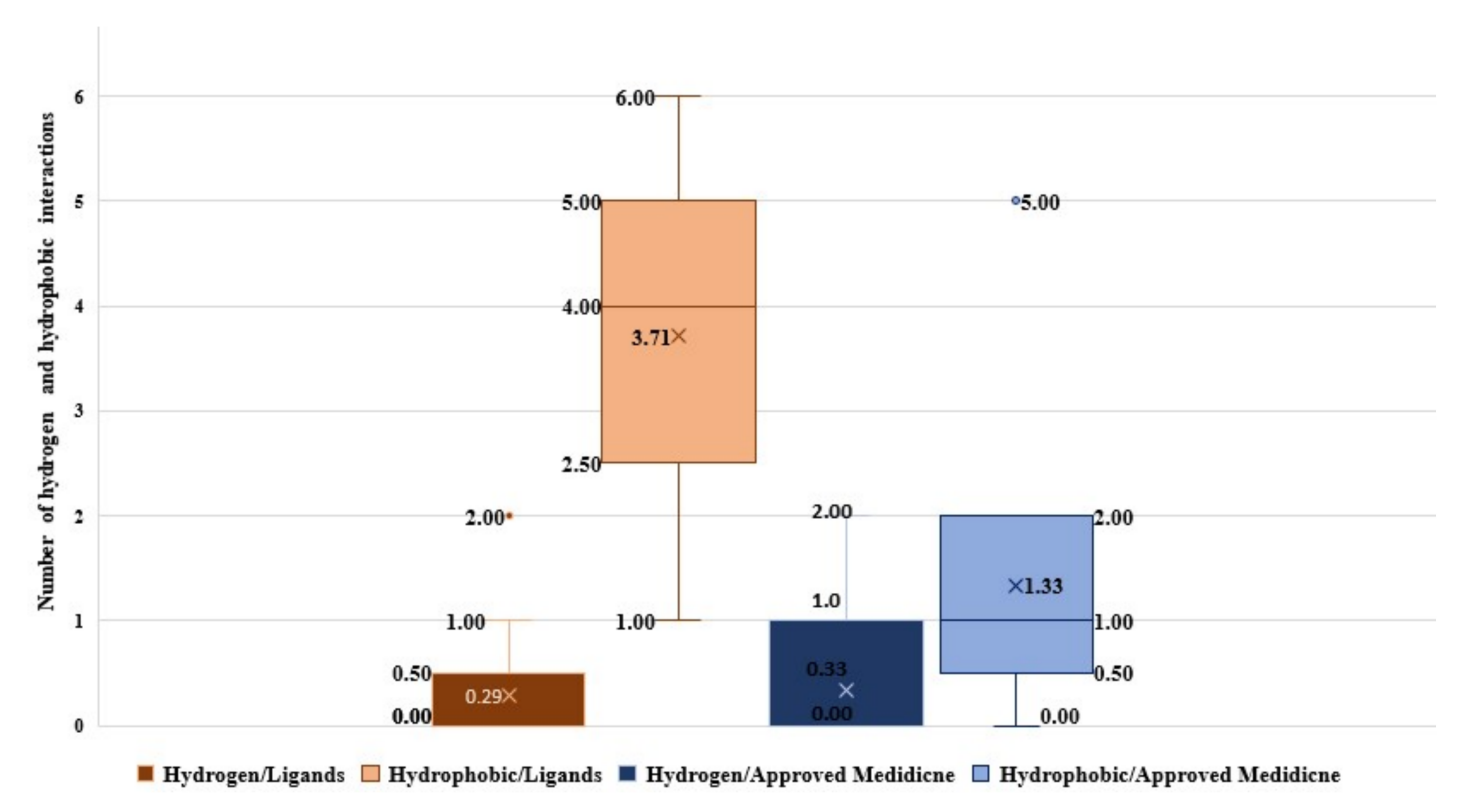
Box plot representing the comparison between hydrogen and hydrophobic bonds among the amino acid residues of the COVID-19 M^pro^ substrate-binding pocket produced by docking with the six newly synthesized ligands **3a–f**, Remdesivir, and Molnupiravir.

The number of hydrogen bonds varied between the ligands **3a–f** and the two approved medicine docked with the COVID-19 M^pro^ pocket, with a mean of 0.29 + 1.34 and 0.33 + 5.80, respectively (Figure 5). The maximum number of hydrogen interactions was 2 in ligands **3a–f** and 4 for the approved drugs (Figure 6). All compounds in this study displayed H-bonds. The hydrophobic interactions for the different amino acids varied between ligands **3a–f** and the approved medicines (Figure 6). The minimum hydrophobic interactions were 0 for both, whereas the maximum hydrophobic interactions were 6 and 5 for ligands **3a–f** and the approved medication, respectively (Figure 5).

The nine docking poses of ligands **3a–f** and the approved drugs Remdesivir and Molnupiravir showed an excellent fitting with the COVID-19 M^pro^ substrate pocket. The mean of the nine poses for each ligand was analyzed and compared using one-way ANOVA and Tukey’s pairwise comparison. The binding affinity readings of the nine poses were significantly varied (Table 1).

**Figure 7:**
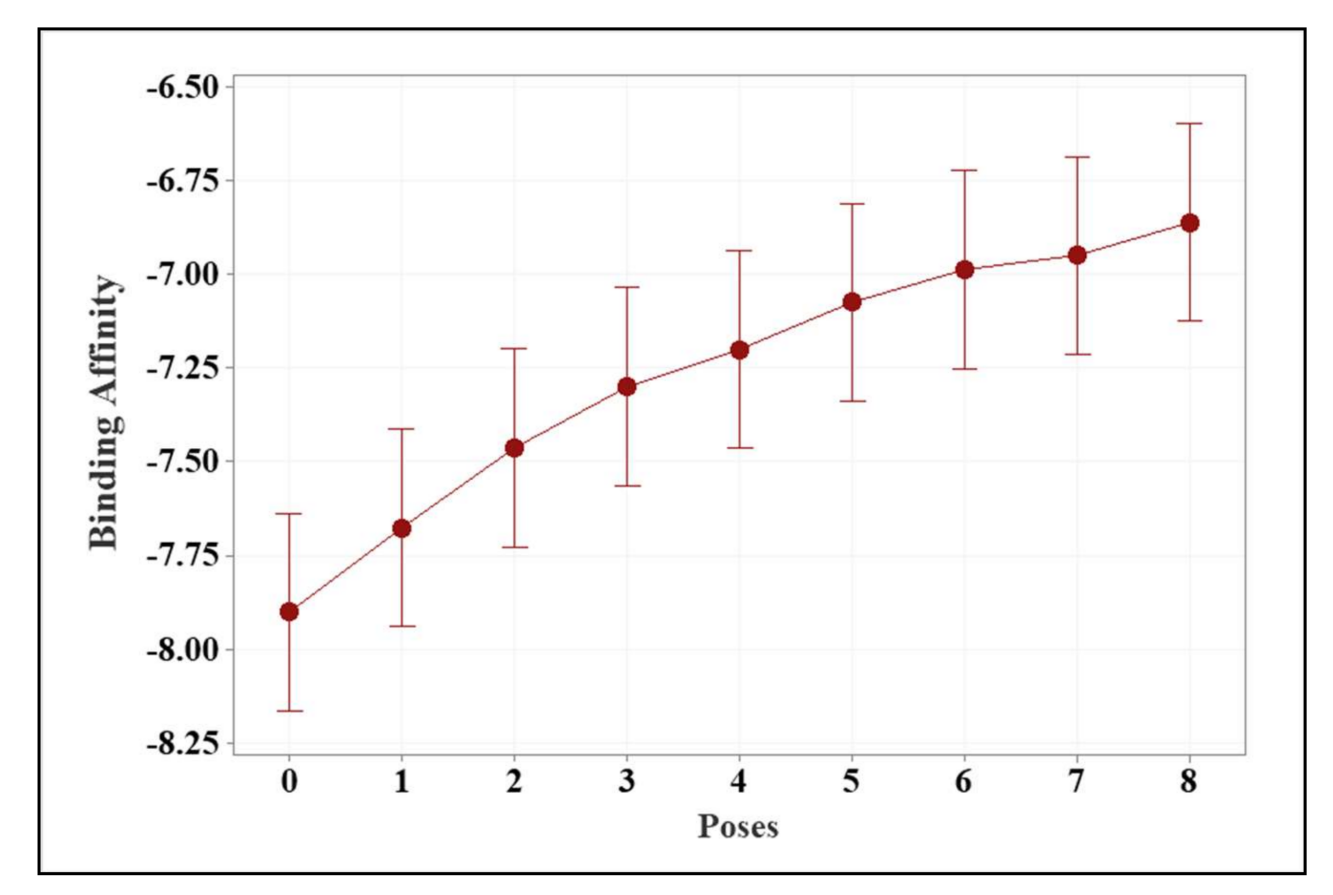
Interval plot of the binding affinities of the nine poses. The interval plots for the binding affinities of the synthesized ligands and the approved drugs were blotted for the interval calculated using a pooled standard division at a confidence level of 95%.

The variation was shown clearly in the interval plot, which demonstrated a negative relationship between the pose number and the binding affinity. The highest binding affinity was recorded for pose 0, using the pooled STDEV of ±0.37 (Figure 7).

Significant differences using ANOVA (*p*-value) was 0.00, which was lower than 0.05 (Table 1). The Tukey pairwise comparison analysis also showed the detailed variation of the binding affinity readings as a mean of those of the nine poses between the ligands and the approved drugs (Table 2 and Figure 7).

Compound **3f** had the highest binding affinity with an average binding energy (−7.81 ± 0.48 kcal/mol) with no significant differences at a *p*-value of 0.05 compared with other ligands, except **3c** and the approved drugs (Table 2). All the ligands recorded higher binding affinities than the approved drugs (Table 2). However, ligands **3e** (average binding energy: 7.25 ± 0.46 kcal/mol) and **3c** (average binding energy: 7.13 ± 0.46 kcal/mol), which had the lowest binding affinity in comparison with the other ligands, had no significant variation with Remdesivir and Molnupiravir (Table 2).

### 2.2. In Silico Toxicity Prediction for Compounds **3a–f**

The ProTox-II server used to detect toxicity of the synthesized compounds **3a–f**. It predicts possible toxicity for a potential chemical to be used as a drug [29,30]. The results showed that the LD_50_ (mg/kg) in parallel with the toxicity class for **3a–f** and the approved medicine as a comparison. Ligands **3a–d** were classified into toxic class 3 with LD_50_ (300 mg/kg). Ligands **3e** and **3f** and the approved medicines (Remdesivir and Molnupiravir) were predicted to be under toxic class 4 with the first three having an LD_50_ of 1000 mg/kg and Molnupiravir having an LD_50_ of 826 mg/kg. The average LD_50_ values, for the six ligands and the approved drugs, were similar (43.51 ± 1.60 mg/kg). They all had the same prediction accuracy of 54.26% (Table 3).

The ProTox-II web server was used to predict organ toxicity (hepatotoxicity). Compounds **3a–f** and Molnupiravir were expected to have weak activities, behaving as toxic compounds to hepatot, but no activity was expected from Remdesivir. Ligand **3b** was noticed to have the highest carcinogenic and mutagenic probabilities of 0.77 and 0.75, respectively. However, ligand **3d**, Molnupiravir, and Remdesivir were predicted to have no carcinogenic and mutagenic activity. Ligands **3a**, **3c**, **3e**, and **3f** were found to have weak carcinogenic and mutagenic activities. All ligands, as well as the approved drugs, had no immunotoxicity with a probability of 0.99. Interestingly, the ligands and the approved drugs behaved similarly with an average hepatotoxicity activity probability of 0.74 + 0.08 (Table 4).

### 2.3. Drug Likeness Evaluation

SwissADME was used to predict the possibility of using ligands **3a–f** as drugs by evaluating physicochemical properties, drug-likeness, and pharmacokinetics [39,40,41]. The same analysis was also carried out for Molnupiravir and Remdesivir. The physicochemical properties evaluated by ADMT included the molecular weight (g/mol), the molecular refractivity, and the topological polar surface area (Å^2^). Table 5 summarizes the physiochemical properties, lipophilicity, and the drug-likeness evaluation. The molecular weight ranged between 368.45 and 429.45 g/mol for the ligands, which was higher than that of Molnupiravir (329.31 g/mol) and lower than that of Remdesivir (602.58 g/mol) (Table 5). All ligands, including Molnupiravir, had drug-likeness bioavailability ranging between 150 and 500 g/mol. The total surface area polarity (TSAP) was in the expected range of 20 and 130 Å^2^. The TSAPs of most ligands **3a**, **3c**, and **3d** were 78.21 Å^2^, and the TSAPs of **3e** and **3f** were also similar at 87.44 Å^2^. However, the TSAP of **3b** was 144.26 Å^2^ closer to that of Molnupiravir (143.14 Å^2^), whereas Remdesivir showed the most significant value (213.36 Å^2^). The solubility (H should not be higher than six for those molecules that might be used as drugs). Ligands 4–7 had H-Bond acceptor (HBA). **3a**, **3c**, and **3d** had four HBAs, **3e** and **3b** had five HBAs, and **3b** had seven HBAs. All ligands **3a–c** had lower HBAs than Molnupiravir and Remdesivir, which were 8 and 12, respectively.

**Figure 8:**
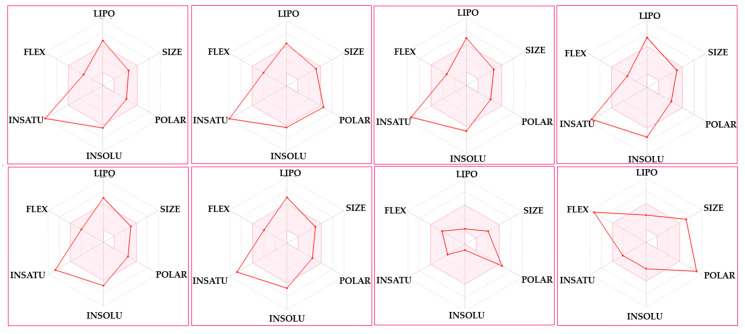
Bioavailability radar for ligands **3a–f** and the approved drugs Molnupiravir and Remdesivir.

The bioavailability radar of the six ligands was drawn based on the physicochemical properties, lipophilicity, size, polarity, solubility, unsaturation, and flexibility (Figure 8). The optimal range of these properties is pink, whereas the red line represents ligands and approved drugs properties. As shown in Figure 8, all ligands fell entirely for size, polarity, and flexibility, except **3b** which was slightly out for polarity. All the six ligands were slightly out for polarity and solubility; however, they were all out for the unsaturation fractions property (Figure 8). Molnupiravir was entirely in the pink area and slightly out for polarity like **3b**. Thus, the bioavailability radar for Remdesivir showed that it fell in the red area at lipophilicity, solubility, and unsaturation. It is out of the other three— properties size, polarity, and flexibility. The six ligands fulfilled some properties of drug-likeness, but not all. All ligands, except **3b**, were predicated to be orally bioavailable and flexible with a suitable size (Figure 8). SwissADME was also used to evaluate the pharmacokinetics properties of ligands **3a–f**. There was no P-glycoprotein (P-gp) substrate for all of them, except **3b** and Remdesivir, justifying their good intestinal absorption. The bioavailability exhibited low gastrointestinal (GI) absorption and the blood–brain barrier (BBB) permeant for all of them, including both medicines (Table 6). The study’s components interacted with one or two isoenzymes of the cytochrome P (CYP) family, confirming their better effectiveness with little toxicity, which was most similar to Molnupiravir (Table 6).

Importantly, when applying the Lipinski rule of five (ROF5), i.e., a molecular weight of <500 g/mol, a logP value of <5, the number of H-bond donors of <5, the number of H-bond acceptors of <10 [42], and an additional rule proposed by Veber concerning the number of the rotatable bonds of <10 [43], all the six ligands were likely to be bioavailable as drugs. However, some were better than others when summing oral bioavailability rules, toxicity, and docking. For example, **3f** had the highest binding affinity when docked to the M^pro^ of SARS-CoV-2, and it also met the ROF5. This can also be applied to the other ligands; thus, the poorest ligand was **3c**, with the lowest binding affinity in its docking and the least extent to which it met the ROF5.

## 3. Materials and Methods

The requisite starting materials such as acetophenone, iodine, and thiourea used to prepare 2-amino-4-phenylthiazoles [44,45,46] were procured from Sigma-Aldrich without any further purification. All the solvents were purified and dried by a standard method.

### 3.1. Instruments

An electrothermal Gallenkamp apparatus was operated to measure the melting points for the newly synthesized compounds. A Pye-Unicam SP300 instrument in potassium bromide discs was used to measure IR spectra. A Varian Mercury VXR-300 spectrometer (300 MHz for ^1^H-NMR and 75 MHz for ^13^C-NMR) was manipulated to measure the ^1^H-NMR and ^13^C-NMR spectra, and the chemical shifts were related to those of the solvent. GCMS-Q1000-EX Shimadzu and GCMS 5988-A HP spectrometers were used to record the mass spectra of the samples at an ionizing voltage of 70 eV. Elemental analyses were carried out at the Microanalytical Centre of Cairo University, Giza, Egypt.

### 3.2. Synthesis of 2-Amino-4-phenyl-5-arylazothiazoles

A cold solution of 2-amino-4-phenylthiazoles (5 mmol) in ethanol (30 mL), buffered with sodium acetate trihydrate, was added to the aryldiazonium chloride prepared by diazotizing appropriate aromatic amines such as aniline, 4-nitroaniline, 4-toluidine, and 3-toluidine dissolved in concentrated hydrochloric acid (6N, 2 mL) with a sodium nitrite solution (0.07 g, 1 mmol) in water (2 mL) [44,45,46]. The addition was carried out portion-wise with stirring at 0–5 °C for 30 min. After the complete addition, the reaction mixture was stirred for a further 4 h at room temperature, then kept in a refrigerator for 12 h and finally diluted with water. The precipitated solid was collected by filtration, washed with water, dried and finally recrystallized from ethanol to afford the corresponding 2-amino-4-phenyl-5-arylazothiazoles (**1**) [44,45,46].

### 3.3. General Procedure for the Synthesis of Compounds **3a–f**

A solution of 2-amino-4-phenyl-5-arylazothiazole (**1**) (1 mmol of each) in ethanol containing aromatic aldehydes (1 mmol) and a few drops of piperidine was refluxed for 3 h and then left to cool. The crude product was collected by filtration and recrystallized from ethanol to generate compounds **3a–f**.

*1-Phenyl-N-[4-phenyl-5-(phenylazo)thiazol-2-yl)methanimine* (**3a**), Dark red crystals (0.31 g, 85%); mp 195–197 °C; IR (KBr) *υ* = 1610 (C=N), 1445 (N=N), cm^−1^; ^1^H-NMR (DMSO-*d_6_*, 300 MHz): *δ* = 7.31–8.32 (15H, m, Ar–H), 9.41 (1H, s, CH=); ^13^C-NMR (DMSO-*d_6_*, 75 MHz): *δ* = 120.3, 126.1, 126.9, 128.1, 128.7, 128.9, 129.2, 129.7, 130.9, 131.4, 132.9, 133.4, 135.3, 140.6, 142.4, 170.3; EIMS *m*/*z* (%): 368 [M^+^] (50), 105 (100), 77 (80). Anal. calcd for C_22_H_16_N_4_S (368.11): C, 71.72; H, 4.38; N, 15.21; S, 8.70. Found: C, 71.66; H, 4.28; N, 15.32; S, 8.64.

*1-(4-Hydroxyphenyl)-N-[4-phenyl-5-((4-nitrophenyl)azo)thiazol-2-yl)methanimine* (**3b**), Brown powder (0.34 g, 80%); mp 212–214 °C; IR (KBr) *υ* = 1602 (C=N), 1446 (N=N), cm^−1^; ^1^H-NMR (DMSO-*d_6_*, 300 MHz): *δ* = 7.31–7.99 (13H, m, Ar–H), 9.42 (1H, s, CH=), 12.31 (1H, s, OH); ^13^C-NMR (DMSO-*d_6_*, 75 MHz): *δ* = 120.4, 126.1, 126.6, 126.7, 128.1, 128.2, 128.9, 129.1, 132.1, 133.4, 134.9, 135.3, 138.3, 140.2, 149.4, 166.1; EIMS *m*/*z* (%): 429 [M^+^] (20), 105 (100), 77 (60). Anal. calcd for C_22_H_15_N_5_O_3_S (429.09): C, 61.53; H, 3.52; N, 16.31; S, 7.47. Found: C, 61.66; H, 3.38; N, 16.42; S, 7.62.

*1-Phenyl-N-[4-phenyl-5-((4-methylphenyl)azo)thiazol-2-yl)methanimine* (**3c**), Brown powder (0.32 g, 84%); mp 150–152 °C; IR (KBr) *υ* = 1600 (C=N), 1447 (N=N), cm^−1^; ^1^H-NMR (DMSO-*d_6_*, 300 MHz): *δ* = 2.32 (3H, s, Ar-CH_3_), 7.35–8.01 (14H, m, Ar–H), 9.49 (1H, s, CH=); ^13^C-NMR (DMSO-*d_6_*, 75 MHz): *δ* = 16.9 (CH_3_), 116.5. 120.3, 126.6, 126.2, 128.4, 128.9, 129.2, 129.7, 132.1, 132.4, 132.9, 133.4, 139.3, 140.6, 142.4, 166.3; EIMS *m*/*z* (%): 382 [M^+^] (10), 105 (100), 77 (80). Anal. calcd for C_23_H_18_N_4_S (382.13): C, 72.23; H, 4.74; N, 14.65; S, 8.38. Found: C, 72.36; H, 4.68; N, 14.42; S, 8.56.

*1-(4-Chlorophenyl)-N-[4-phenyl-5-((4-methylphenyl)azo)thiazol-2-yl)methanimine* (**3d**), Brown powder (0.33 g, 80%); mp 165–167 °C; IR (KBr) *υ* = 1612 (C=N), 1456 (N=N), cm^−1^; ^1^H-NMR (DMSO-*d_6_*, 300 MHz): *δ* = 2.42 (3H, s, Ar-CH_3_), 7.41–8.48 (13H, m, Ar–H), 9.51 (1H, s, CH=); ^13^C-NMR (DMSO-*d_6_*, 75 MHz): *δ* = 16.3 (CH_3_), 113.9, 121.8, 125.4, 126.3, 127.1, 128.9, 129.1, 130.7, 130.8, 137.4, 137.9, 139.3, 141.3, 144.2, 150.4, 164.1; EIMS *m*/*z* (%): 416 [M^+^] (10), 105 (60), 77 (100). Anal. calcd for C_23_H_17_ClN_4_S (416.09): C, 66.26; H, 4.11; N, 13.44; S, 7.69. Found: C, 61.36; H, 4.28; N, 13.52; S, 7.62.

*1-(4-Methoxyphenyl)-N-[4-phenyl-5-((4-methylphenyl)azo)thiazol-2-yl)methanimine* (**3e**), Brown powder (0.31 g, 75%); mp 173–175 °C; IR (KBr) *υ* = 1600 (C=N), 1456 (N=N), cm^−1^; ^1^H-NMR (DMSO-*d_6_*, 300 MHz): *δ* = 2.44 (3H, s, Ar-CH_3_), 4.42 (3H, s, OCH_3_), 7.54–8.07 (13H, m, Ar–H), 9.46 (1H, s, CH=); ^13^C-NMR (DMSO-*d_6_*, 75 MHz): *δ* = 15.8 (CH_3_), 66.2 (OCH_3_), 119.8, 120.3, 123.9, 126.3, 127.5, 128.9, 129.4, 130.7, 132.8, 134.1, 135.4, 138.1, 139.3, 141.3, 143.6, 166.1; EIMS *m*/*z* (%): 412 [M^+^] (10), 105 (100), 77 (50). Anal. calcd for C_24_H_20_N_4_OS (412.14): C, 69.88; H, 4.89; N, 13.58; S, 7.77. Found: C, 70.06; H, 4.99; N, 13.72; S, 7.92.

*1-(4-Methoxyphenyl)-N-[4-phenyl-5-((3-methylphenyl)azo)thiazol-2-yl)methanimine* (**3f**), Brown powder (0.31 g, 75%); mp 222–224 °C; IR (KBr) *υ* = 1600 (C=N), 1457 (N=N), cm^−1^; ^1^H-NMR (DMSO-*d_6_*, 300 MHz): *δ* = 2.42 (3H, s, Ar-CH_3_), 4.47 (3H, s, OCH_3_), 7.30–8.06 (13H, m, Ar–H), 9.49 (1H, s, CH=); ^13^C-NMR (DMSO-*d_6_*, 75 MHz): *δ* = 15.6 (CH_3_), 63.8 (OCH_3_), 114.6, 116.5, 120.1, 120.3, 127.9, 128.3, 128.5, 128.9, 129.4, 131.7, 132.2, 132.5, 132.8, 135.4, 139.5, 140.3, 143.6, 166.1; EIMS *m*/*z* (%): 412 [M^+^] (10), 105 (100), 77 (70). Anal. calcd for C_24_H_20_N_4_OS (412.14): C, 69.88; H, 4.89; N, 13.58; S, 7.77. Found: C, 70.09; H, 4.73; N, 13.33; S, 7.58.

### 3.4. Docking In Silico Studies

The docking calculations of compounds **3a–f**, Molnupiravir, and Remdesivir using 7BQY (Mpro, PDB code: 7BQY; resolution: 1.7 Å) were accomplished using the Autodock Vina wizard in PyRx 0.8. [47]. Settings were made identical for docking in this research study: grid box center_x = 5.37343529609, center_y = 0.301235653306, center_z = 23.0783947527, size_x = 19.9069566462, size_y = 23.5833091318, and size_z = 25.0. The remaining parameters were used as a default setting in the Autodock Vina-PyRx. All drugs and ligands were converted to the SDF file type using Chem. The draw program was used as an input to Autodock vina in PyRx. Energy minimization parameters used for all ligands in this study, before docking were as following: force field = uff; optimization algorithm = conjugate gradients; total number of steps = 200; number of steps = 1; stop if the energy difference is less than 0.1. The proteases (PDB code: 7BQY) were saved in the PDB format after deleting the water molecules and ligands using Discovery Studio Visualizer v17.2.0.16349. The PyMOL molecular viewer was used to present the output data [29]. The schematic diagrams of protein–ligand interactions were generated using the LIGPLOT program [48].

### 3.5. Statistical Analysis

Analysis of variance of one factor (one-way ANOVA) was used as a statistical model to evaluate the binding affinities of both the synthesized ligands (**3a–f**) and the two approved drugs, Molnupiravir and Remdesivir. In addition, Tukey pairwise comparison was applied following one-way ANOVA analysis to group means considering *p* of ≤0.05 as a statistically significant level. ANOVA statistical analyses were carried out using Minitab 19 (www.minitab.com, accessed on 6 November 2021). Boxplot analysis was carried using Excel in Microsoft Office.

### 3.6. In Silico Toxicity Prediction

In silico methods and the ProTox-II platform was used to predict the toxicity levels of the ligands synthesized in this study, which were compared to those of the approved drugs Molnupiravir and Remdesivir [29,30]. The ProTox-II system has five classification steps: (1) acute toxicity (oral toxicity model and six toxicity classes); (2) organ toxicity with one model; (3) toxicological endpoints with four models; (4) toxicological pathways with 12 models; and (5) toxicity targets with 15 models. A total of 33 models were used in this prediction process allowing the prediction for various toxicity endpoints such as hepatotoxicity, cytotoxicity, carcinogenicity, mutagenicity, and immunotoxicity. The Acute oral toxicity predictions for substances were classified into different toxicity classes, depending upon the LD_50_ (mg/kg body weight). These classes were the same class as in the Globally Harmonized System (GHS) classification and labeling of chemicals. These classes were as following: class 1—fatal if swallowed (LD_50_ ≤ 5 mg/kg); class 2—fatal if swallowed (5 mg/kg < LD_50_ ≤ 50 mg/kg); class 3—toxic if swallowed (50 mg/kg < LD_50_ ≤ 300 mg/kg); class 4—harmful if swallowed (300 mg/kg < LD_50_ ≤ 2000 mg/kg); class 5— may be harmful if swallowed (2000 mg/kg < LD_50_ ≤ 5000 mg/kg). The average and SD were calculated when needed.

### 3.7. SwissADME Drug-Likeness Properties Prediction

The pharmacokinetic and drug-like properties of the selected compounds were evaluated using SwissADME (absorption, distribution, metabolism, and excretion) descriptors by a SwissADME online server (http://www.swissadme.ch/, accessed on 22 December 2021 as previously reported [39].

## 4. Conclusions

A straightforward procedure was employed to obtain 1-aryl-*N*-[4-phenyl-5-(arylazo)thiazol-2-yl)methanimines (**3a–f**) in a reasonable high yield. A molecular comparative docking study for **3a–f** was calculated with reference to two approved drugs, Molnupiravir and Remdesivir, using 7BQY (M^pro^, PDB code: 7BQY; resolution: 1.7 Å) under identical conditions and using minimum energy towards greener pastures. The results showed more negative (more negative is better) binding scores for **3a–f** than for Molnupiravir and Remdesivir. The high scores of **3a–f** indicated a better fit of the ligands in the receptor’s active amino acid residues of 7BQY. However, the increased binding score was not enough to judge the compounds’ suitability as inhibitors for COVID-19. However, the superpositions of the ligands with respect to the approved drugs, Molnupiravir and Remdesivir, uncovered similar behaviors of both in the amino acid cavity of 7BQY. PASS and Way2drug cytotoxicity tools were used to reveal that **3d** was the same as Molnupiravir, suggesting a need for a further study in the context of potential therapeutic agents for COVID-19. Further analysis using SwissADME was performed to show that the six ligands were likely to be bioavailable drugs.

## Figures and Tables

**Table 1:** One-way ANOVA for the binding affinities of the 9 poses (pose 0 to pose 8).

Source of Variance	DF	F-Value	*p*-Value
Ligands and approved drugs	7	7.42	0.00
Number of poses	8	7.12	0.00

**Table 2:** Means and SDs of the binding affinities of the nine docking poses for compounds against the COVID-19 main protease 7BQY.

Ligand and Approved Medicines		Average Binding Energy (kcal/mol)
Ligands	**3f**	−7.81 ± 0.48 ^a^
	**3d**	−7.52 ± 0.21 ^a,b^
	**3a**	−7.41 ± 0.29 ^a,b,c^
	**3b**	−7.36 ± 0.50 ^a,b,c^
	**3e**	−7.25 ± 0.46 ^a,b,c,d^
	**3c**	−7.13 ± 0.46 ^b,c,d^
Approved medicine	Remdesivir	−6.93 ± 0.33 ^c,d^
	Molnupiravir	−6.71 ± 0.23 ^d^

Notes: Different letters denote statistically significant differences using Tukey’s multiple comparison test at *p* ≤ 0.05.

**Table 3:** Acute oral toxicity predicted by the ProTox-II web server for ligands **3a–f** and the approved drugs.

Ligand	Oral Toxicity Prediction Results			
	Predicted LD_50_ (mg/kg)	Predicted Toxicity Class	Average Similarity (%)	Prediction Accuracy (%)
**3a**	300	3	44.61	54.26
**3b**	300	3	42.28	54.26
**3c**	300	3	44.32	54.26
**3d**	300	3	45.74	54.26
**3e**	1000	4	43.73	54.26
**3f**	1000	4	42.98	54.26
**Molnupiravir**	1000	4	40.93	54.26
**Remdesivir**	826	4	79.41	69.26

**Table 4:** Organ toxicity and toxicological endpoints-predicted activity calculated using the ProTox-II web server for ligands **3a–f**, Molnupiravir, and Remdesivir.

Ligands	Hepatotoxicity		Carcinogenicity		Immunotoxicity		Mutagenicity		Cytotoxicity	
	Activity	Probability	Activity	Probability	Activity	Probability	Activity	Probability	Activity	Probability
**3a**	Yes	0.58	Yes	0.59	No	0.99	Yes	0.57	No	0.80
**3b**	Yes	0.55	Yes	0.77	No	0.99	Yes	0.75	No	0.76
**3c**	Yes	0.55	Yes	0.60	No	0.99	Yes	0.58	No	0.82
**3d**	Yes	0.57	No	0.59	No	0.99	No	0.55	No	0.82
**3e**	Yes	0.58	Yes	0.57	No	0.99	Yes	0.63	No	0.73
**3f**	Yes	0.58	Yes	0.57	No	0.99	Yes	0.63	No	0.73
**Molnupiravir**	Yes	0.56	No	0.50	No	0.96	No	0.50	No	0.74
**Remdesivir**	No	0.56	No	0.55	No	0.90	No	0.62	No	0.55

**Table 5:** Physicochemical properties and lipophilicity of ligands **3a–f** in comparison with the approved drugs Molnupiravir and Remdesivir.

Physicochemical	3a	3b	3c	3d	3e	3f	Molnupiravir	Remdesivir
**Molecular weight (g/mol)**	368.45	429.45	382.48	416.93	412.51	412.51	329.31	602.58
**Heavy atom**	27	31	28	29	30	30	23	42
**Arom. heavy atom**	23	23	23	23	23	23	6	15
**Fraction Csp3**	0.00	0.00	0.04	0.04	0.08	0.08	0.62	0.48
**Rotatable bond**	5	6	5	5	6	6	6	14
**H-bond acceptor**	4	7	4	4	5	5	8	12
**H-bond donor**	0	1	0	0	0	0	4	4
**Molar refractivity**	111.86	122.71	116.83	121.84	123.32	123.32	76.02	150.43
**Polar surface area (Å^2^)**	78.21	144.26	78.21	78.21	87.44	87.44	143.14	213.36
**Lipophilicity**								
**MLOGP**	4.05	2.57	4.27	4.74	3.92	3.92	−1.15	0.18
**WLOGP**	6.98	6.59	7.28	7.94	7.29	7.29	−1.65	2.21
**XLOGP3**	6.36	5.83	6.72	7.35	6.69	6.69	−1.34	1.91
**Drug-likeness evaluation**								
**Lipinski**	0	0	1	1	0	0	0	2

**Table 6:** The pharmacokinetics properties of ligands **3a–f** in comparison with those of the approved drugs, Molnupiravir and Remdesivir.

Pharmacokinetics		3a	3b	3c	3d	3e	3f	Molnupiravir	Remdesivir
**Gastrointestinal (GI) absorption**		Low	Low	Low	Low	Low	Low	Low	Low
**Blood–brain barrier (BBB) permeant**		No	No	No	No	No	No	No	No
**P-glycoprotein (P-gp) substrate**		No	No	No	Yes	No	No	No	Yes
**Inhibitor**	**CYP1A2**	Yes	No	No	No	No	No	No	No
	**CYP2C19**	Yes	No	Yes	No	No	No	No	No
	**CYP2C9**	Yes	Yes	Yes	No	Yes	Yes	Yes	No
	**CYP2D6**	No	No	No	No	No	No	No	No
	**CYP3A4**	No	No	No	No	No	No	No	Yes

## Data Availability

The Supplementary Data has been in a separate sheet.

## Supplementary Materials

Figure S1: IR of compound (**3a**), Figure S2: ^1^H-NMR of compound (**3a**), Figure S3: ^13^C-NMR of compound (**3a**), Figure S4: MS of compound (**3a**), Figure S5: IR of compound (**3e**), Figure S6: ^1^H-NMR of compound (**3e**), Figure S7: ^13^C-NMR of compound (**3e**), Figure S8: MS of compound (**3e**).

## Sample Availability

Samples of the compounds are available from the authors.

## References

[1] Borbone N., Piccialli G., Roviello G.N., Oliviero G. (2021). Nucleoside Analogs and Nucleoside Precursors as Drugs in the Fight against SARS-CoV-2 and Other Coronaviruses. Molecules.

[2] Singh T.U., Parida S., Lingaraju M.C., Manickam K., Kumar D., Singh R.K. (2020). Drug repurposing approach to fight COVID-19. Pharmacol. Rep..

[3] Gao Z., Xu Y., Sun C., Wang X., Guo Y., Qiu S., Ma K. (2021). A systematic review of asymptomatic infections with COVID-19. J. Microbiol. Immunol. Infect..

[4] Yüce M., Filiztekin E., Özkaya K.G. (2021). COVID-19 diagnosis—A review of current methods. Biosens. Bioelectron..

[5] Li Y., Tenchov R., Smoot J., Liu C., Watkins S., Zhou Q. (2021). A Comprehensive Review of the Global Efforts on COVID-19 Vaccine Development. ACS Cent. Sci..

[6] Noor R. (2021). A Review on the Effectivity of the Current COVID-19 Drugs and Vaccines: Are They Really Working Against the Severe Acute Respiratory Syndrome Coronavirus 2 (SARS-CoV-2) Variants?. Curr. Clin. Microbiol. Rep..

[7] Costanzo M., De Giglio M.A.R., Roviello G. (2021). Anti-Coronavirus Vaccines: Past Investigations on SARS-CoV-1 and MERS-CoV, the Approved Vaccines from BioNTech/Pfizer, Moderna, Oxford/AstraZeneca and others under Development Against SARS-CoV-2 Infection. Curr. Med. Chem..

[8] Sultana J., Mazzaglia G., Luxi N., Cancellieri A., Capuano A., Ferrajolo C., de-Waure C., Ferlazzo G., Trifiro G. (2020). Potential effects of vaccinations on the prevention of COVID-19: Rationale, clinical evidence, risks, and public health considerations. Exp. Rev. Vacc..

[9] Germain R.N., Meier-Schellersheim M., Nita-Lazar A., Fraser I.D.C. (2011). Systems biology in immunology: A computational modeling perspective. Annu. Rev. Immunol..

[10] Roviello G., Roviello V., Autiero I., Saviano M. (2016). Solid phase synthesis of TyrT, a thymine–tyrosine conjugate with poly(A) RNA-binding ability. RCS Adv..

[11] Afzal O., Kumar S., Haider M.R., Ali M.R., Kumar R., Jaggi M., Bawa S. (2015). A review on anticancer potential of bioactive heterocycle quinoline. Eur. J. Med. Chem..

[12] Ali S.H., Sayed A.R. (2021). Review of the synthesis and biological activity of thiazoles. Synth. Commun..

[13] Altamimi M.A., Hussain A., Alshehri S., Imam S.S., Alnami A., Bari A. (2020). Novel hemocompatible imine compounds as alternatives for antimicrobial therapy in pharmaceutical application. Processes.

[14] Bashiri M., Jarrahpour A., Rastegari B., Iraji A., Irajie C., Amirghofran Z., Malek-Hosseini S., Motamedifar M., Haddadi M., Zomorodian K. (2020). Synthesis and evaluation of biological activities of tripodal imines and β-lactams attached to the 1,3,5-triazine nucleus. Monatsh. Chem..

[15] Da Silva E.T., Araújo A.S., Moraes A.M., de Souza L.A., Lourenço M.C.S., de Souza M.V.N., Wardell J.L., Wardell S.M.S.V. (2016). Synthesis and Biological Activities of Camphor Hydrazone and Imine Derivatives. Sci. Pharm..

[16] Sakthinathan S.P., Suresh R., Kamalakkannan D., Mala V., Sathiyamoorthi K., Vanangamudi G., Thirunarayanan G. (2018). Microwave assisted synthesis, spectral correlation and antimicrobial Evaluation of some aryl imines. J. Chil. Chem. Soc..

[17] Khan M.S., Siddiqui S.P., Tarannum N. (2017). A systematic review on the synthesis and biological activity of hydrazide derivatives. Hygeia. J. Drugs Med..

[18] Jeelani A., Muthu S., Narayana B. (2021). Molecular structure determination, Bioactivity score, Spectroscopic and Quantum computational studies on (E)-N′-(4-Chlorobenzylidene)-2-(napthalen-2-yloxy) acetohydrazide. J. Mol. Struct..

[19] Alsaygh A., Al-Humaidi J., Al-Najjar I. (2014). Synthesis of Some New Pyridine-2-yl-Benzylidene-Imines. Int. J. Org. Chem..

[20] Donnelly R., Patrinos H.A. (2021). Learning loss during COVID-19: An early systematic review. Prospects.

[21] Marin B.G., Aghagoli G., Lavine K., Yang L., Siff E.J., Chiang S.S., Salazar-Mather T.P., Dumenco L., Savaria M.C., Aung S.N. (2021). Predictors of COVID-19 severity: A literature review. Rev. Med. Virol..

[22] Zhang X., Tan Y., Ling Y., Lu G., Liu F., Yi Z., Jia X., Wu M., Shi B., Xu S. (2020). Viral and host factors related to the clinical outcome of COVID-19. Nature.

[23] Jin Z., Du X., Xu Y., Deng Y., Liu M., Zhao Y., Zhang B., Li X., Zhang L., Peng C. (2020). Structure of Mpro from SARS-CoV-2 and discovery of its inhibitors. Nature.

[24] Vallianou N.G., Tsilingiris D., Christodoulatos G.S., Karampela I., Dalamaga M. (2021). Anti-viral treatment for SARS-CoV-2 infection: A race against time amidst the ongoing pandemic. Metab. Open.

[25] Nguyen H.L., Thai N.Q., Truong D.T., Li M.S. (2020). Remdesivir Strongly Binds to Both RNA-Dependent RNA Polymerase and Main Protease of SARS-CoV-2: Evidence from Molecular Simulations. J. Phys. Chem. B.

[26] Van Gunsteren W.F., Karplus M. (1982). Protein Dynamics in Solution and in a Crystalline Environment: A Molecular Dynamics Study. Biochemistry.

[27] Musumeci D., Ullah S., Ikram A., Roviello G.N. (2021). Novel insights on nucleopeptide binding: A spectroscopic and In Silico investigation on the interaction of a thymine-bearing tetrapeptide with a homoadenine DNA. J. Mol. Liq..

[28] Wolinski K., Hinton J.F., Wishart D.S., Sykes B.D., Richards F.M., Pastone A., Hayes D.M. Hyper Chem Version 8.pdf PYRX, USA 2001. http://www.swissadme.ch/.

[29] Drwal M.N., Banerjee P., Dunkel M., Wettig M.R., Preissner R. (2014). ProTox: A web server for the in-silico prediction of rodent oral toxicity. Nucleic Acids Res..

[30] Banerjee P., Eckert A.O., Schrey A.K., Preissner R. (2018). ProTox-II: A webserver for the prediction of toxicity of chemicals. Nucleic Acids Res..

[31] DeLano W.L. (2004). PyMOL Reference Guide.

[32] Rubin D., Chan-Tack K., Farley J., Sherwat A. (2020). FDA Approval of Remdesivir—A Step in the Right Direction. N. Engl. J. Med..

[33] Shah B., Modi P., Sagar S.R. (2020). In silico studies on therapeutic agents for COVID-19: Drug repurposing approach. Life Sci..

[34] Alsafi M.A., Hughes D.L., Said M.A. (2020). First COVID-19 molecular docking with a chalcone-based compound: Synthesis, single-crystal structure and Hirshfeld surface analysis study. Acta Crystallogr. Sect. C Struct. Chem..

[35] Imran M., Alshrari A.S., Asdaq S.M.B., Abida (2021). Trends in the development of remdesivir based inventions against COVID-19 and other disorders: A patent review. J. Infect. Public Health.

[36] Pardo J., Shukla A.M., Chamarthi G., Gupte A. (2020). The journey of remdesivir: From Ebola to COVID-19. Drugs Context.

[37] Yoon J.J., Toots M., Lee S., Lee M.E., Ludeke B., Luczo J.M., Ganti K., Cox R.M., Sticher Z.M., Edpuganti V. (2018). Orally Efficacious Broad-Spectrum Ribonucleoside Analog Inhibitor of Influenza and Respiratory Syncytial Viruses. Antimicrob. Agents Chemother..

[38] Fischer W., Eron J.J., Holman W., Cohen M.S., Fang L., Szewczyk L.J., Sheahan T.P., Baric R., Mollan K.R., Wolfe C.R. (2021). Molnupiravir, an Oral Antiviral Treatment for COVID-19. Preprint. medRxiv.

[39] Daina A., Michielin O., Zoete V. (2017). SwissADME: A free web tool to evaluate pharmacokinetics, druglikeness and medicinal chemistry friendliness of small molecules. Sci. Rep..

[40] Parlak C., Alver Ö., Ouma C.N.M., Rhyman L., Ramasami P. (2021). Interaction between favipiravir and hydroxychloroquine and their combined drug assessment: In silico investigations. Chem. Pap..

[41] Ritchie T.J., Ertl P., Lewis R. (2011). The graphical representation of ADME-related molecule properties for medicinal chemists. Drug Discov. Today.

[42] Lipinski C.A., Lombardo F., Dominy B.W., Feeney P.J. (2001). Experimental and computational approaches to estimate solubility and permeability in drug discovery and development settings. Adv. Drug Deliv. Rev..

[43] Veber D.F., Johnson S.R., Cheng H.-Y., Smith B.R., Ward K.W., Kopple K.D. (2002). Molecular Properties That Influence the Oral Bioavailability of Drug Candidates. J. Med. Chem..

[44] Yen M.S. (2012). Synthesis and solvatochromism of heterocyclic bichromophoric dyes derived from 2-aminothiazole. Pigment. Resin Tech..

[45] Derkowska-Zielinska B., Skowronski L., Biitseva A., Grabowski A., Naparty M.K., Smokal V., Kysil A., Krupka O. (2017). Optical characterization of heterocyclic azo dyes containing polymers thin films. Appl. Surf. Sci..

[46] Prajapati A.K., Modi V.P. (2011). Synthesis and biological activity of n-{5-(4-methylphenyl) diazenyl-4-phenyl-1, 3-thiazol-2-yl}benzamide derivatives. Quim. Nova.

[47] Trott O., Olson A.J. (2009). AutoDock Vina: Improving the speed and accuracy of docking with a new scoring function, efficient optimisation, and multithreading. J. Comput. Chem..

[48] Wallace A.C., Laskowski R.A., Thornton J.M. (1995). Ligplot: A program to generate schematic diagrams of protein-ligand interactions. Protein Eng. Des. Sel..

