# Supplementary material for: Potential COVID-19 Drug Candidates Based on Diazinyl-Thiazol-Imine Moieties: Synthesis and Greener Pastures Biological Study
Source: Molecules. 2022 Jan 13;27(2):488. doi: 10.3390/molecules27020488 (PMC8777737; doi:10.3390/molecules27020488)

Figure S1: IR of compound (**3a**)

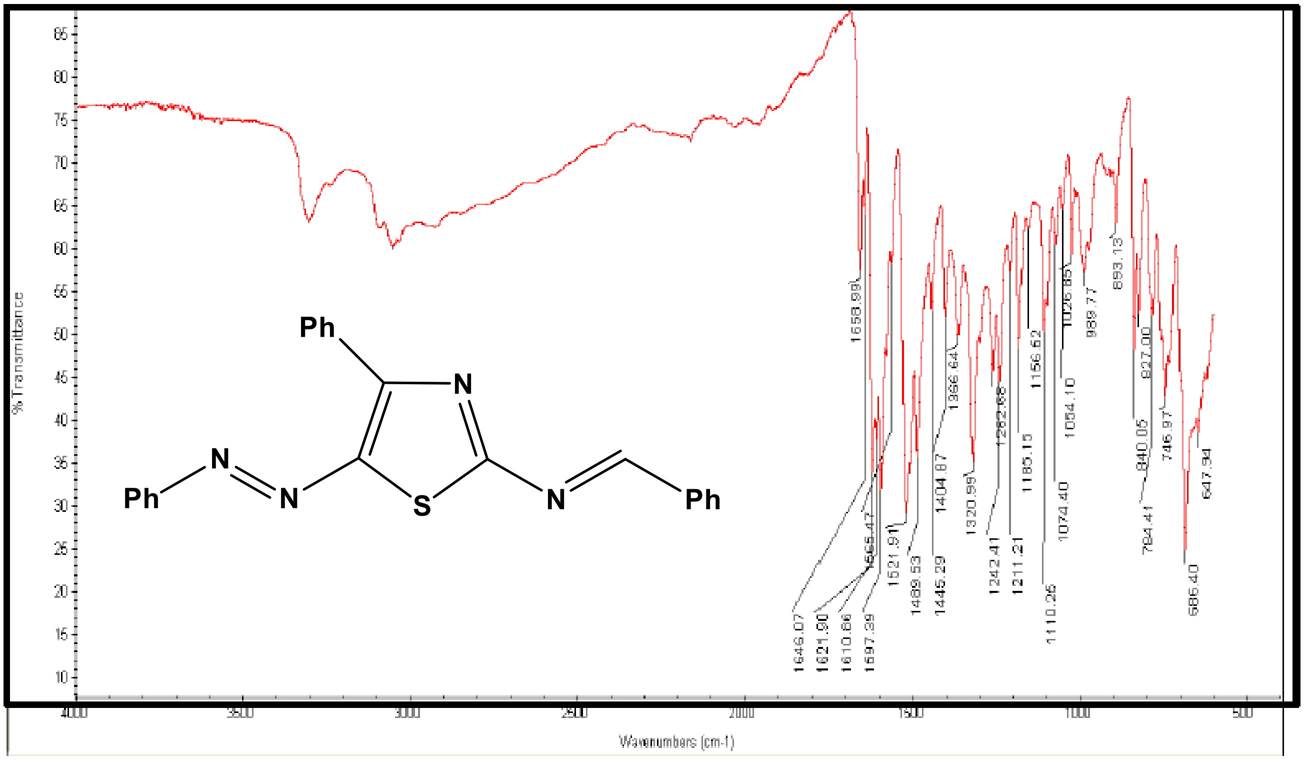

Figure S2: ^{1}H NMR of compound (**3a**)

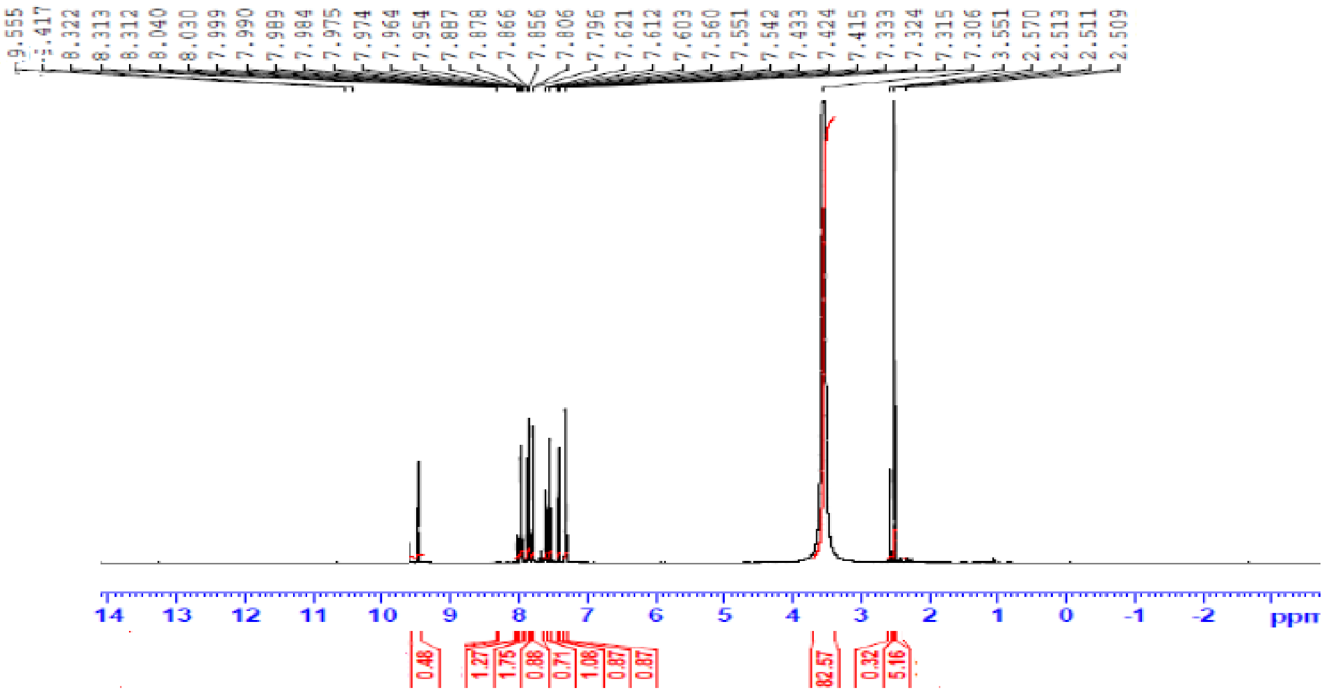

Figure S3: ^{13}C NMR of compound (**3a**)

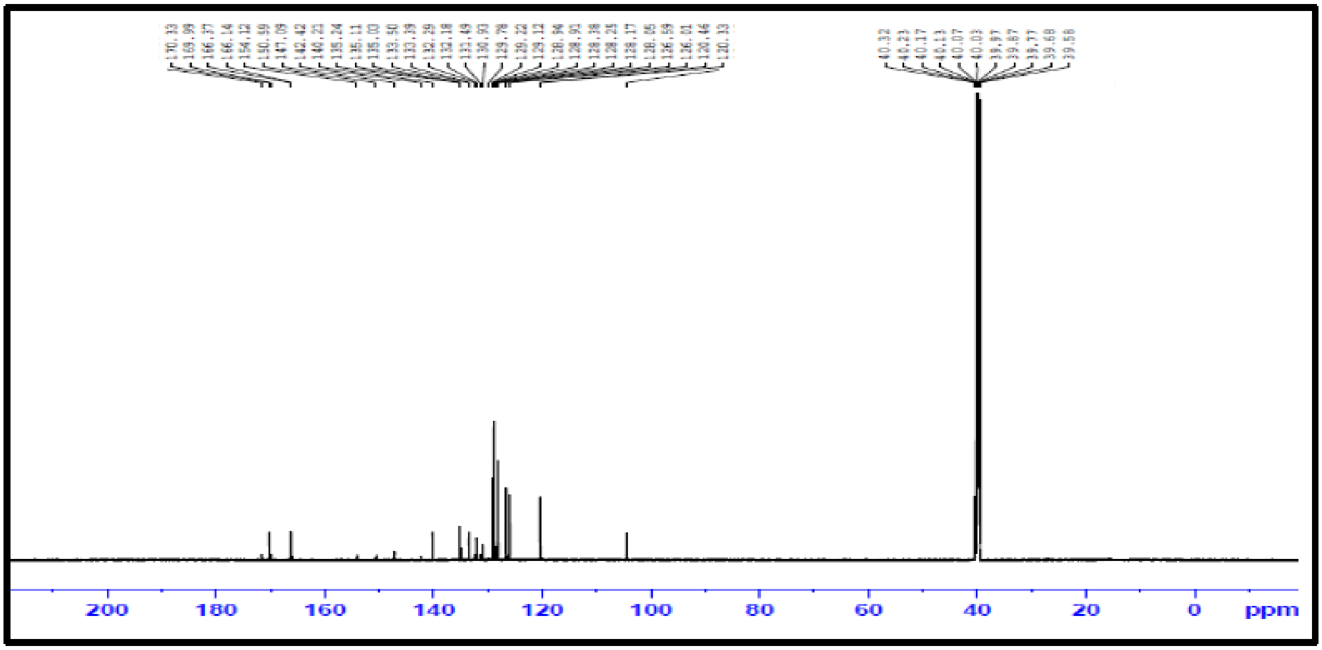

Figure S4: MS of compound (**3a**)

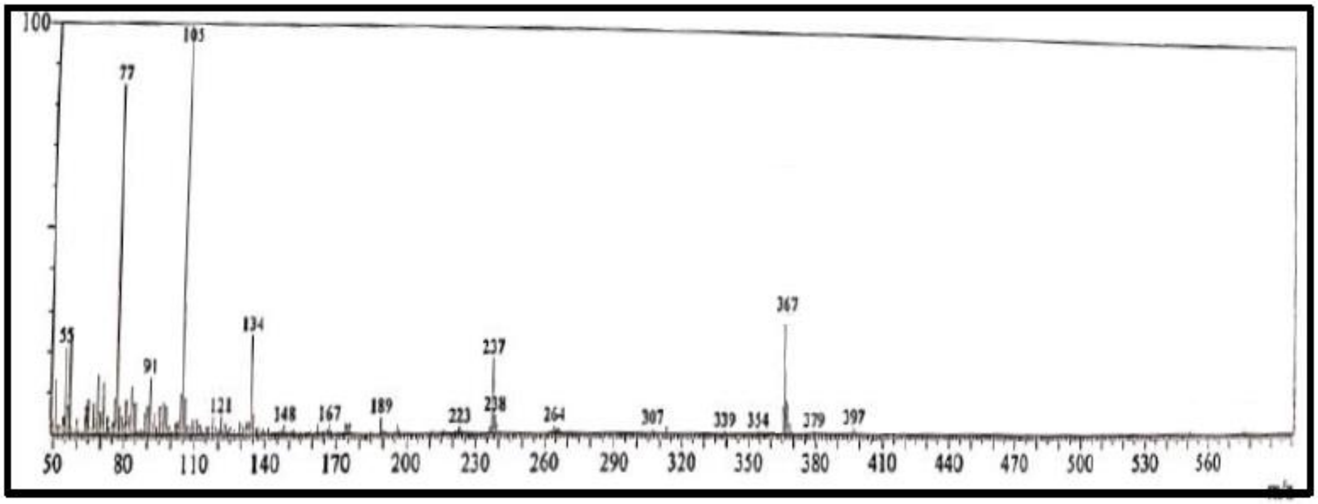

Figure S5: IR of compound (**3e**)

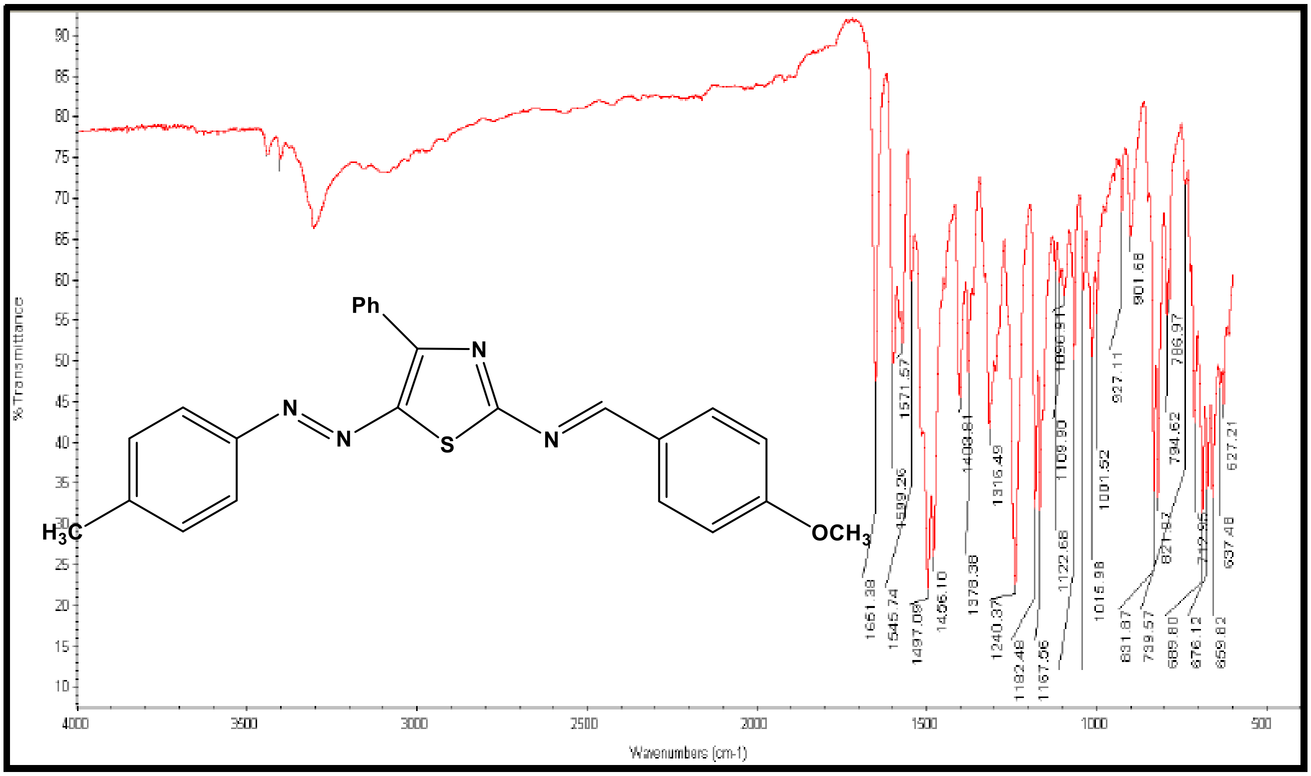

Figure S6: ^{1}H NMR of compound (**3e**)

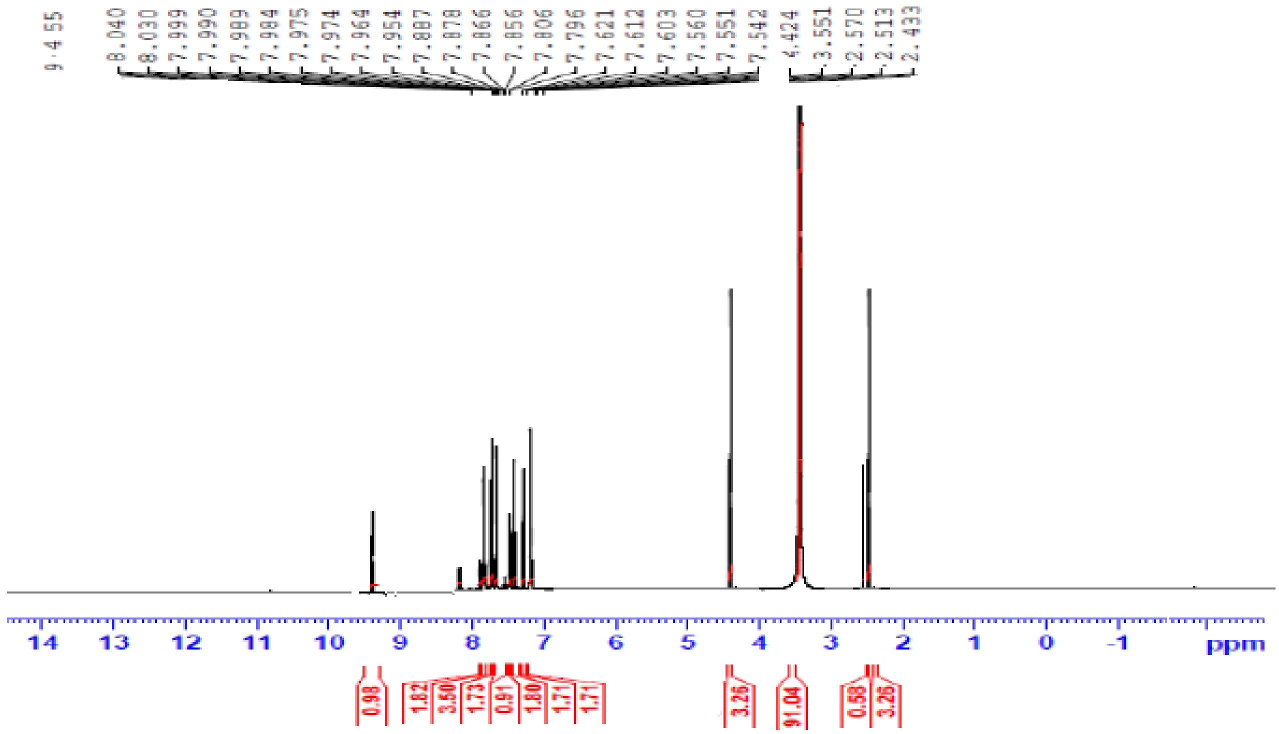

Figure S7: ^{13}C NMR of compound (**3e**)

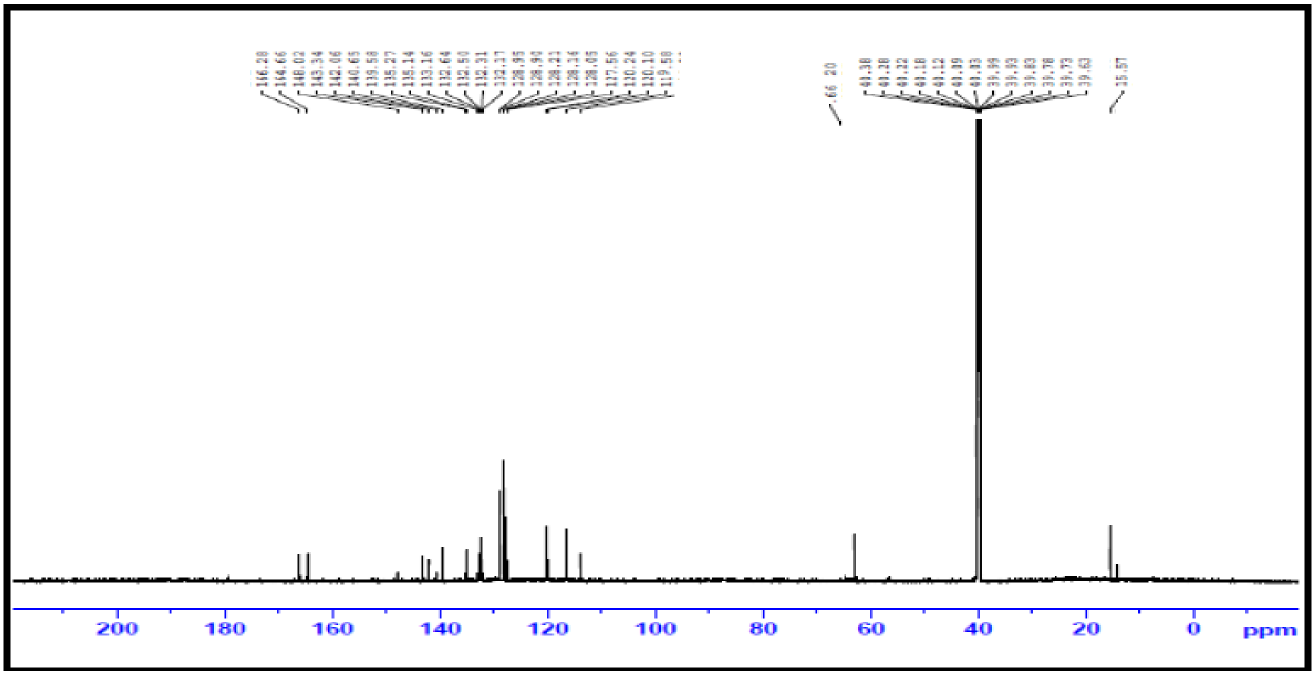

Figure S8: MS of compound (**3e**)

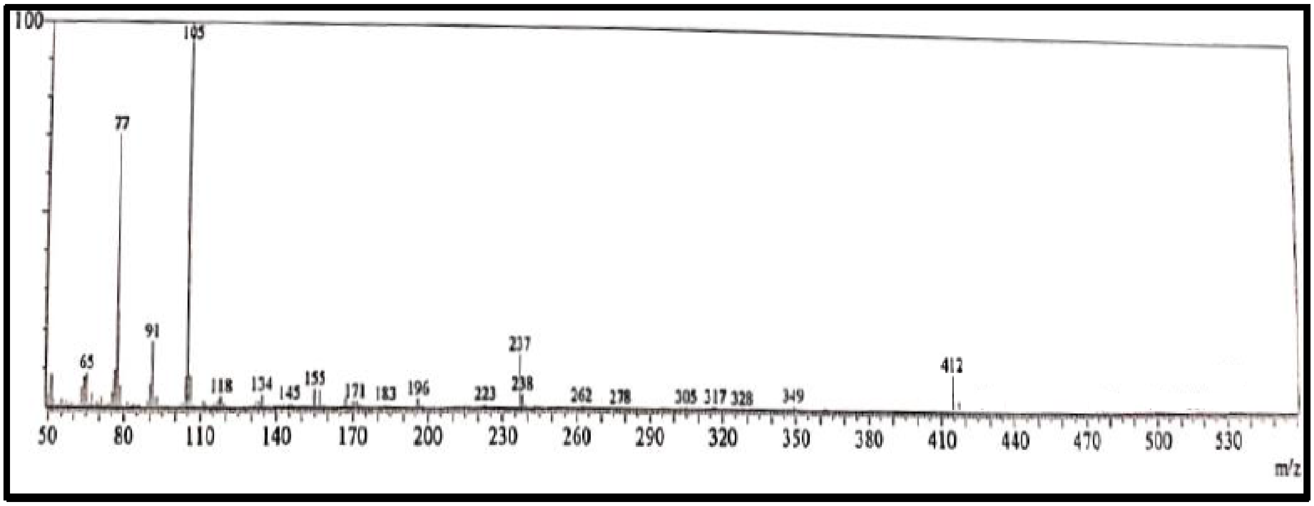

Supplement: Supplementary file 1 [file molecules-27-00488-s001.zip › molecules-1511011-supplementary.pdf]
